# Recurrent Transverse Colon Volvulus After Operative Detorsion: A Case Report

**DOI:** 10.7759/cureus.52419

**Published:** 2024-01-17

**Authors:** Gemechu Lemi Yadeta, Birhanu Abdisa Tesso, Langa James Oriho

**Affiliations:** 1 Department of Surgery, Jimma University, College of Public Health and Medical sciences, Jimma, ETH

**Keywords:** recurrent volvulus, operative detortion, colonic volvulus, large bowel obstruction, transverse colon volvulus

## Abstract

Transverse colon volvulus is a rare type of colonic volvulus. Here, we present a case of a 40-year-old male patient with a recurrent transverse colon volvulus after operative detorsion. He presented with a history of intermittent crampy abdominal pain of three days duration associated with failure to pass both feces and flatus. He has a history of abdominal distention and vomiting. The patient has a history of repeated abdominal surgeries. His last surgery was two years before the presentation, laparotomy with operative detorsion without colopexy for viable transverse colon volvulus. The patient was explored, and transverse colectomy was done with two-stage procedures. The transverse colon volvulus can occur simultaneously or metachronously with other types of colonic volvulus. A high index of suspicion is needed for diagnosis. Management of transverse colon volvulus should be resection with or without primary anastomosis.

## Introduction

Transverse colon volvulus is a rare condition [1]. It was first described in 1932 by Kallio in Scandinavian literature [1,2]. Risk factors of transverse colon volvulus include non-fixation of the colon and chronic constipation with megacolon [1-3]. Transverse colon volvulus typically presents with signs and symptoms of bowel obstruction. The radiographic investigations include X-rays in which the appearance of the transverse colon volvulus resembles the sigmoid volvulus. Computerized tomography (CT) scans have replaced other radiological investigations because they can differentiate pathologies [4]. Management includes colonoscopic or operative detorsion associated with high recurrence, and the best option is emergent exploration and resection. We present a 40-year-old male patient with a recurrent transverse colon volvulus after operative detorsion.

## Case presentation

A 40-year-old male patient presented to the emergency department with a history of intermittent crampy abdominal pain for three days duration that was associated with failure to pass both feces and flatus. He has a history of abdominal distention and multiple episodes of vomiting of ingested matter. His regular diet was high-fiber food; otherwise, he was not a smoker or alcoholic.

He had a history of repeated abdominal surgeries. His first surgery was 11 years ago, in which laparotomy and Hartman’s procedure (sigmoid colectomy and end colostomy) were done for gangrenous sigmoid volvulus, and the second surgery was colostomy reversal was done after three months from the index surgery. His third surgery was done three years before the presentation, in which laparotomy with adhesiolysis was done for postoperative adhesions, and the fourth surgery was done two years before the presentation in which laparotomy with operative detorsion without colopexy for viable transverse colon volvulus, which was intraoperatively diagnosed after he presented with large bowel obstruction symptoms. He was then given an appointment for follow-up, but unfortunately, he was lost to follow-up.

On presentation, he was sick, and his vitals were a blood pressure of 109/82 millimeters of mercury (mmHg), pulse rate of 86 beats per minute, respiratory rate of 20 breaths per minute, and temperature of 36 degrees Celsius. He had a slightly dry tongue and buccal mucosa. The abdomen was distended with visible peristalsis, there was a midline laparotomy scar and left lower quadrant stoma closure scar, the bowel sounds were hyperactive, hyper tympanic percussion note, there was mild tenderness in the periumbilical area, and there was formed stool on the rectum but no blood or mass. With an impression of large bowel obstruction secondary to recurrent transverse colon volvulus, he was resuscitated with 2 liters of normal saline and produced adequate urine output.

**Figure 1:**
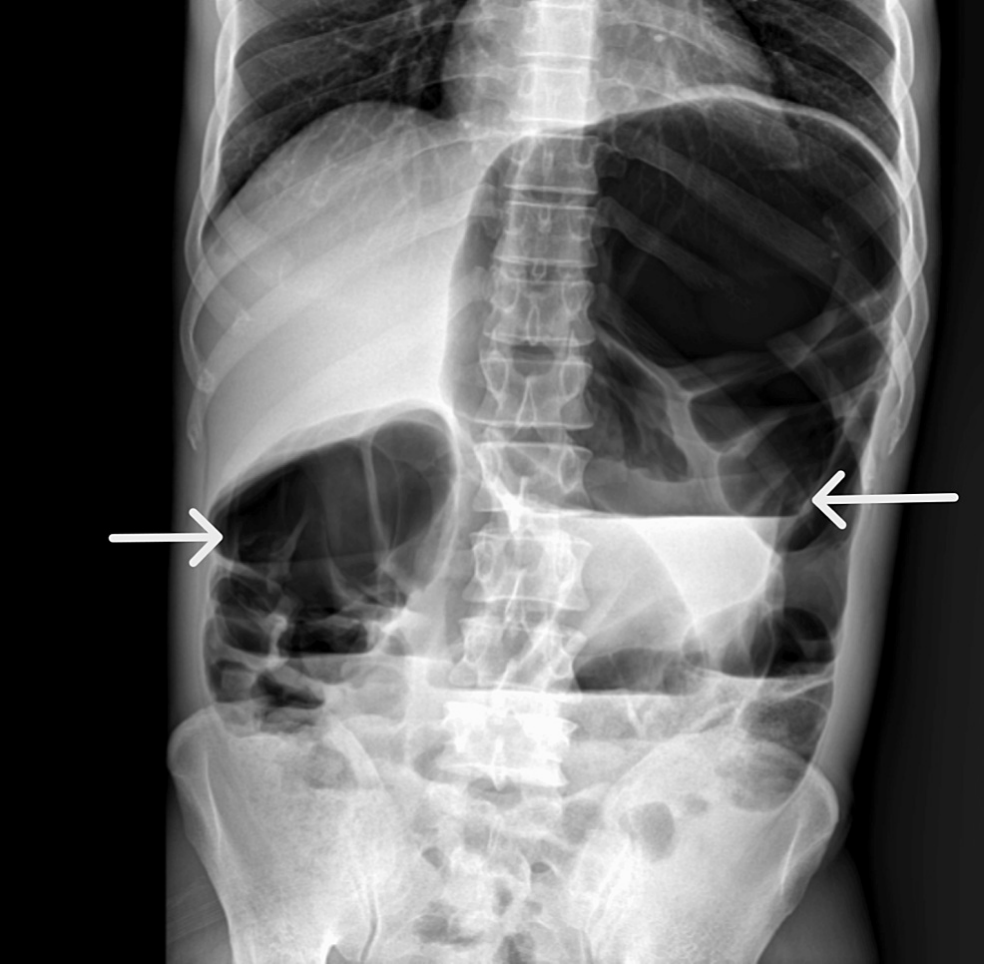
Distended large bowel loops (white arrows) depicting large bowel obstruction

He was investigated with an erect abdominal X-ray, and the X-ray showed dilated large bowel loops, as seen in Figure 1. Also, laboratory investigations were done, as seen in Table 1. He had an increased neutrophil percentage with increased monocytes and basophil count; otherwise, the other parameters were normal.

**Table 1:** Laboratory data (H): higher than normal values; (L): lower than normal values; uL: microliter; mcL: microliter; g/dL: grams per deciliter; U/L: units per liter; mg/dL: milligrams per deciliter; mEq/L: milliequivalent per liter

Variable	Reference Range	On Presentation
White-cell count (10³/uL)	3.00-15.00	8.52
Differential count		
Neutrophils (10³/mcL)	1.50-7.00	6.73
Neutrophils %	37.0-72.0	78 (H)
Lymphocytes (10³/mcL)	1.0-3.70	0.79
Monocytes (10³/mcL)	0.0-0.70	0.95 (H)
Eosinophils (10³/mcL)	0.0-0.40	0.03
Basophils (10³/mcL)	0.0-0.10	0.2 (H)
Platelet Count (10³/uL)	150-450	322
Red-Cell Count (10⁶/mcL)	2.50-5.50	5.25
Hemoglobin (g/dL)	8.0-17.0	16
Hematocrit (%)	26.0-50.0	47.3
Alanine aminotransferase (U/L)	0.0-41	9.3
Aspartate aminotransferase (U/L)	0.0-40	21.3
Alkaline phosphatase (U/L)	40-129	76
Creatinine (mg/dl)	0.7-1.20	0.70
Sodium (mEq/L)	136-145	144
Potassium (mEq/L)	3.5-5.1	3.35
Chloride (mEq/L)	98-107	103.4

**Figure 2:**
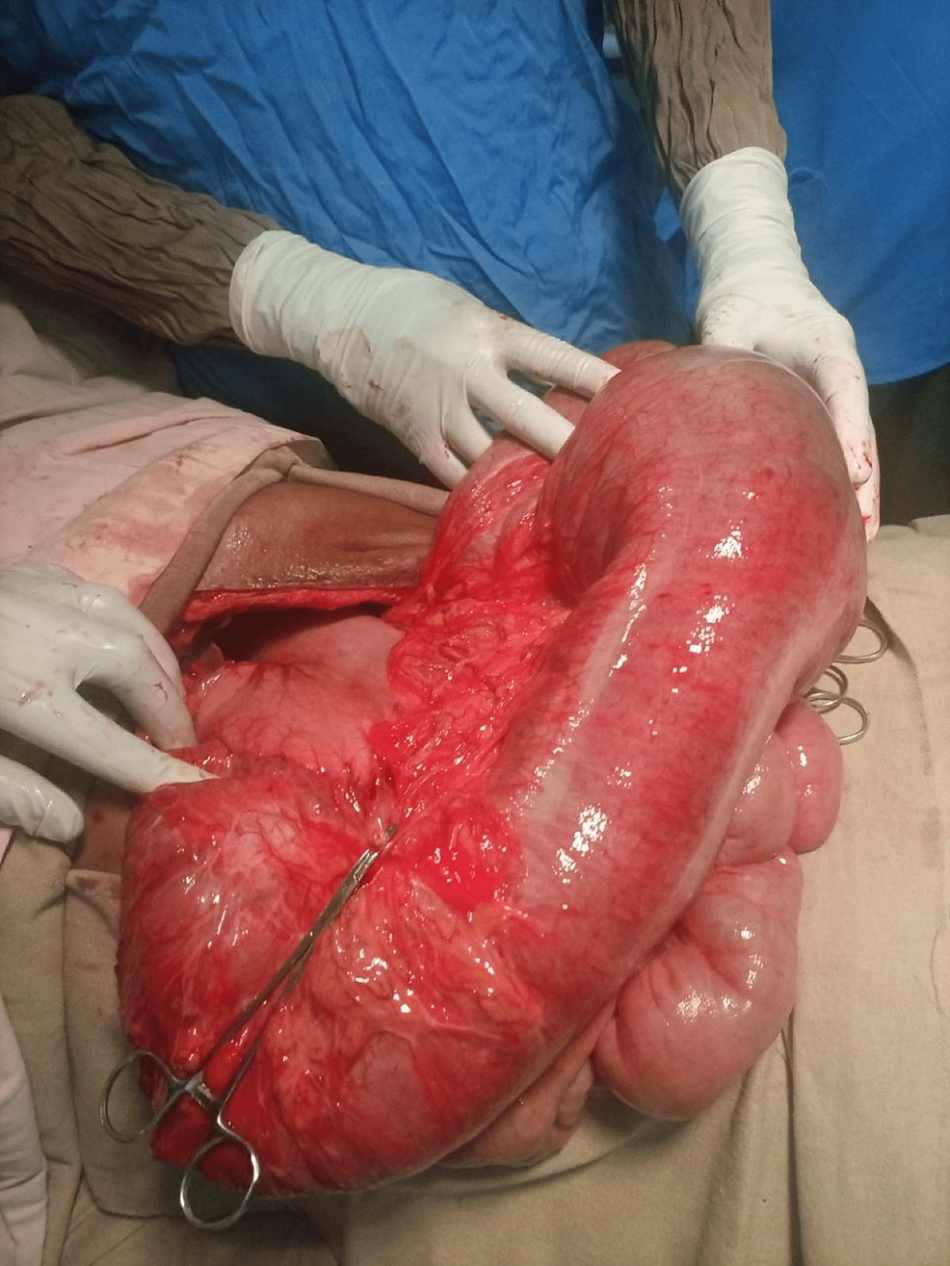
Intraoperative picture of viable transverse colon volvulus

Subsequently, he was taken to the operating room, and an emergency laparotomy was done. Intraoperatively, there was a viable dilated redundant transverse colon twisted 180 degrees clockwise on its mesentery, as seen in Figure 2, and there was a band from the mid-transverse colon to the right lower abdominal wall. There was no sigmoid colon (resected). The band was released, and around 1-meter transverse colon was resected. It was challenging to do primary anastomosis because there was tension between the two ends of the colon, and the bowel was grossly distended. The ascending colon was mobilized as an end colostomy in the right upper quadrant, and the descending colon was mobilized as a mucous fistula in the left lower quadrant. The abdomen was closed, and the stoma was matured. Postoperatively, he was put on antibiotics and analgesia, started a liquid diet, and tolerated food on day 3. Subsequently, the patient was discharged with an appointment on day 6.

**Figure 3:**
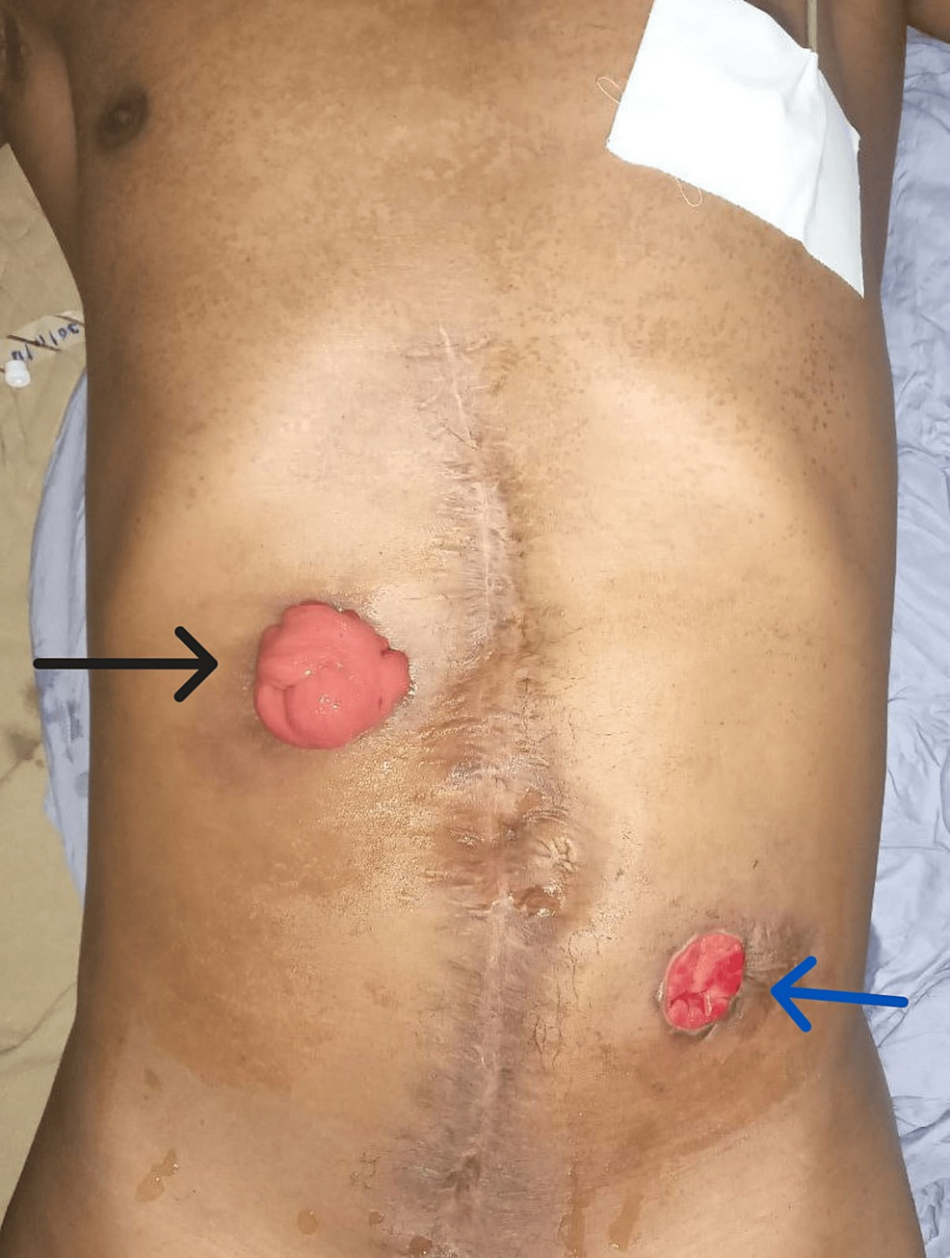
Intraoperative picture before colostomy reversal depicting colostomy (black arrow) and mucous fistula (blue arrow)

He was admitted four months after the discharge for elective stoma reversal surgery. Bowel preparation was done before the surgery. Intraoperatively, the colostomy and the mucous fistula were brought down, as seen in Figure 3. There were inter-loop adhesions, the ascending and the descending colon were mobilized, and end-to-end colo-colic anastomosis was done. Postoperatively, the patient was on analgesia, started the liquid diet on the third postop, and tolerated feeding on the fourth postop. He was discharged with an appointment after 15 days of hospital stay. His follow-up visits after two weeks and one month were uneventful.

## Discussion

Colonic volvulus is one of the causes of large bowel obstruction. It represents up to 13% to 42% of all intestinal obstructions in Africa, South America, Russia, Eastern Europe, the Middle East, India, and Brazil, the so-called “volvulus belt” areas. In North America, Western Europe, and Australia, colonic volvulus represents less than 5% of all intestinal obstruction [3]. The most common site in which the volvulus occurs in the colon is the sigmoid colon (61%-75%), cecum (15%-34.5%), transverse colon (3%-5%), and splenic flexure (2%) [4]. In normal conditions, the anatomic attachments create broad fixation of the transverse colon and flexures, preventing torsion [4].

Risk factors for developing transverse colon volvulus included congenital absence of normally colonic attachments like Chilaiditi syndrome or acquired conditions that contribute to colonic distention and mesenteric lengthening and atypical segments of the colon are free to undergo torsion [4,5]. Risk factors can be intestinal malrotation, an enlarged colon, a long mesentery, Hirschsprung disease, pregnancy, abdominal adhesions, chronic constipation, Clostridium difficile-associated pseudomembranous colitis, and high fiber diet [4,5]. Our patient had a previous history of sigmoid volvulus before developing transverse colon volvulus, a history of small bowel obstruction secondary to postoperative adhesion, and a history of transverse colon volvulus in which operative dertorsion was done. Previous abdominal surgeries can cause bowel concrescence or bowel translocation [5]. In addition, Ethiopia is one of the countries of the Horn of Africa that resides in the Volvulus belt, and a stable Ethiopian diet contains high fiber, which is one of the risk factors for developing transverse colon volvulus [6]. All the mentioned risk factors predisposed our patient to colonic volvulus. It is rare for the transverse colon volvulus to occur simultaneously with other colonic volvulus. Still, there is literature in which transverse colon volvulus can simultaneously occur with sigmoid volvulus, transverse colon volvulus with cecal volvulus, transverse colon volvulus with ascending colon volvulus, or transverse to descending colon volvulus and megacolon with mesenterium commune [4,6-9]. Due to the risk factors of colonic volvulus, in some patients, the transverse colon volvulus can also occur after sigmoid colon resection (metachronous colonic volvulus), the duration of the sigmoid resection usually a month to four years before presentation with transverse colon volvulus [10,11]. In our patient, transverse colon volvulus occurred 11 years after sigmoid colectomy.

Transverse colon volvulus can present as either acute fulminating or subacute progressive. The presentation was first categorized by Eisenstat et al. The acute fulminating presents with bowel infraction, peritonitis, and marked leukocytosis. The subacute type presents with massive abdominal distension, mild abdominal pain without rebound tenderness, nausea, or vomiting, and normal leukocyte count secondary to the lack of ischemia at early stages or mildly elevated [4,11]. Our patient presentation was subacute progressive with normal vitals and was a recurrence transverse volvulus. Physical findings and imaging modalities aid in the diagnosis of transverse colon volvulus. An X-ray can show a coffee bean sign, a northern sign, or an inverted U-shaped sign. A CT scan can show whirl signs and differentiate between pathologies like malignancy [4,7]. In our case, the diagnosis was clinically due to a previous type of surgery done for transverse colon volvulus, and the X-ray showed signs of large bowel obstruction.

Treatment of transverse colon volvulus starts with resuscitation, and options of management include detorsion or resection of the transverse colon with primary anastomosis or with stoma formation [12]. Surgical management of transverse colon volvulus can be one- or two-stage procedures [9,13]. In a one-stage procedure, the bowel is resected, and primary anastomosis is done to avoid stoma creation. In a two-stage procedure, the bowel is resected with stoma creation, and the stoma will be reversed after the patient is stable. However, recurrence after resection and primary anastomosis varies between 22% and 36% [13]. Detortion and decompression can be done operatively with or without colopexy, but it is associated with a high recurrence rate [9,13]. Colonoscopic detorsion is not advised for transverse colonic volvulus because of the high recurrence rate [12], and it is difficult and risky for patients with a colon not fixed to the retroperitoneum [9]. The mortality rate for transverse colon volvulus can reach 33% [1].

## Conclusions

Transverse colon volvulus is a rare condition that can occur simultaneously or metachronously with other types of colonic volvulus. A high index of suspicion needed for the diagnosis of transverse colon volvulus if there is identified one of the risk factors. Management of transverse colon volvulus should be resection with or without primary anastomosis. Operative detorsion is associated with a high recurrence rate and should be indicated for patients with viable transverse colon volvulus who cannot tolerate bowel resection surgery.
